# Evolution of Zinc Oxide Nanostructures Grown on Graphene by Ultrasonic Spray Pyrolysis and Its Statistical Growth Modelling

**DOI:** 10.1186/s11671-015-1163-1

**Published:** 2015-11-25

**Authors:** Amgad Ahmed Ali, Abdul Manaf Hashim

**Affiliations:** Malaysia-Japan International Institute of Technology, Universiti Teknologi Malaysia, Jalan Sultan Yahya Petra, 54100 Kuala Lumpur, Malaysia

**Keywords:** Surface response methodology, Graphene oxide, Spray pyrolysis, Zinc oxide, Acetylacetonate, Nanostructure

## Abstract

The evolution of zinc oxide nanostructures grown on graphene by alcohol-assisted ultrasonic spray pyrolysis was investigated. The evolution of structures is strongly depended on pyrolysis parameters, i.e., precursor molarity, precursor flow rate, precursor injection/deposition time, and substrate temperature. Field-effect scanning electron microscope analysis, energy dispersive X-ray spectroscopy, X-ray diffraction, and transmission electron microscopy were used to investigate the properties of the synthesized nanostructures and to provide evidence for the structural changes according to the changes in the pyrolysis parameters. The optimum parameters to achieve maximum density and well-defined hexagonally shaped nanorods were a precursor molarity of 0.2 M, an injection flow rate of 6 ml/min, an injection time of 10 min, and a substrate temperature of 250–355 °C. Based on the experimental results, the response surface methodology (RSM) was used to model and optimize the independent pyrolysis parameters using the Box-Behnken design. Here, the responses, i.e., the nanostructure density, size, and shape factor, are evaluated. All of the computations were performed using the Design-Expert software package. Analysis of variance (ANOVA) was used to evaluate the results of the model and to determine the significant values for the independent pyrolysis parameters. The evolution of zinc oxide (ZnO) structures are well explained by the developed modelling which confirms that RSM is a reliable tool for the modelling and optimization of the pyrolysis parameters and prediction of nanostructure sizes and shapes.

## Background

Graphene, which is a two-dimensional (2D) sheet of *sp*^2^-hybridized carbon, has attracted great attention because of its exceptional optical, electrical, chemical, and mechanical properties, which provide promise for developing new generations of functional nanomaterials for various applications [1–3]. To achieve these targeted applications, there have been significant efforts to control and modify the properties of graphene through various functionalization routes [4]. Much research has been performed to develop semiconducting/graphene hybrid structures either by vapor-phase [5] or liquid-phase techniques [6–8]. In the past few decades, zinc oxide (ZnO) nanostructures have been thoroughly considered in many works for optoelectronic and photovoltaic device applications [9–11]. Recently, it was reported that ZnO/graphene hybrid nanostructures have excellent potential for transparent flexible electrical and optical devices, including flexible photovoltaics, displays, and light emitters [7, 12]. Vapor-phase deposition of ZnO utilizing β-diketonates Zn precursors such as acetylacetonate has been reported as one of the promising routes for growing ZnO nanostructures [9, 11]. Spray pyrolysis technique is a simple and industrially scalable process because of the abundance and stability of the precursors and the low maintenance and setup costs involved in scaling-up the process. Moreover, it allows deposition of homogeneous metal oxide phases with excellent physical properties for several applications. Ultrasonic spray pyrolysis is primarily discussed in relation to film deposition, and very few works have focused on its ability to deposit nanostructures.

In this article, we report the evolution of ZnO nanostructures grown on graphene using a low-temperature ultrasonic-assisted spray pyrolysis technique. The effects of pyrolysis parameters, namely, precursor flow rate (*Q*), molarity (*M*), substrate temperature (*T*), and precursor injection/deposition time (*t*), on the grown structures were investigated. Next, the growth modelling and process optimization were carried out to explain the observed evolution of ZnO nanostructures. The responses, i.e., structure density (*ζ*), structure shape factor (*φ*), and structure size (*r*), were evaluated. Here, the modelling and optimization of the ultrasonic spray pyrolysis parameters for the growth of ZnO nanostructures on a graphene layer using the response surface methodology (RSM) method were performed. RSM is an approach that uses a philosophy of sequential experimentation with the ultimate goal of optimizing a process while using available experimentation resources efficiently. Thus, the use of RSM for optimization purposes reduces the cost of expensive analysis methods and their associated numerical noise [13]. Most of the literature regarding ZnO/graphene hybrid structures has focused on the discussion of their structural morphologies [8, 14–19], and very little of this research has focused for the optimization of the process parameters [20, 21]. To our knowledge, there is no report regarding statistical modelling and subsequent optimization of the growth of ZnO nanostructures on graphene using ultrasonic spray pyrolysis.

## Methods

### Experimental Procedures

Single-layer graphene on a SiO_2_ (285 nm)/Si wafer (Graphene Laboratories, USA) was used as the substrate. Zinc acetylacetonate hydrate powder, Zn(C_5_H_7_O_2_)_2_ xH_2_O (Sigma-Aldrich), was used without any further purification. Other chemicals, such as solvents and reagents, were research grade and used as received. The substrates were cleaned with ethanol and vacuum dried at 60 °C prior to the growth. First, a substrate was heated to the required temperature under 35-mbar vacuum. Then, ZnO liquid source (zinc acetylacetonate in ethanol solution) was supplied to the ultrasonic atomizer at the desired flow rate. Finally, the liquid precursor was sprayed on the substrate surface. Because an ultrasonic nozzle was used to atomize the solution into nano-droplets, the temperature required for vaporizing the droplets was quite low. The experimental conditions are developed based on the L_16_(4^4^) array of RSM and summarized in Table 1. Here, the precursor flow rate, molarity of the liquid precursor, substrate temperature, and precursor injection time were set as the independent parameters, whereas the nanostructure density, shape factor, and structure size were the responses of interest.

**Table 1:** RSM L_16_(4^4^) array of the combinations of parameters for the experimental runs

Run (*R*_i_)	Flow rate (ml/min)	Substrate temperature (°C)	Deposition time (min)	Molarity (M)	Nano rods density (%)	Nanorods shape factor	Nanorods size (Nm)
1	8	500	0.5	0.4	0.36	1	39.2
2	1	575	15.25	0.0	8.79	0.23	35.0
3	1	355	15.25	0.7	31.07	1.1	26.3
4	0.05	500	0.5	0.4	28.03	1	36.4
5	10	355	15.25	0.0	31.06	1.1	32.2
6	0.05	500	30	0.4	19.56	0.57	11.8
7	1	355	38	0.0	76.54	1.1	30.5
8	8	210	0.5	0.4	1.51	1.1	28.0
9	0.05	210	30	0.4	94.17	0.36	10.6
10	0.05	210	0.5	0.4	1.53	0.09	25.2
11	1	355	15.25	0.2	30.92	0.36	29.0
12	0.01	355	15.25	0.0	31.03	1.1	28.0
13	8	500	30	0.4	19.54	0.01	14.6
14	8	210	30	0.4	95.15	0.09	13.4
15	1	135	15.25	0.0	0.15	1	18.2
16	1	355	0.5	0.0	1.05	1	26.6

### Statistical Modelling and Process Optimization

**Fig. 1:**
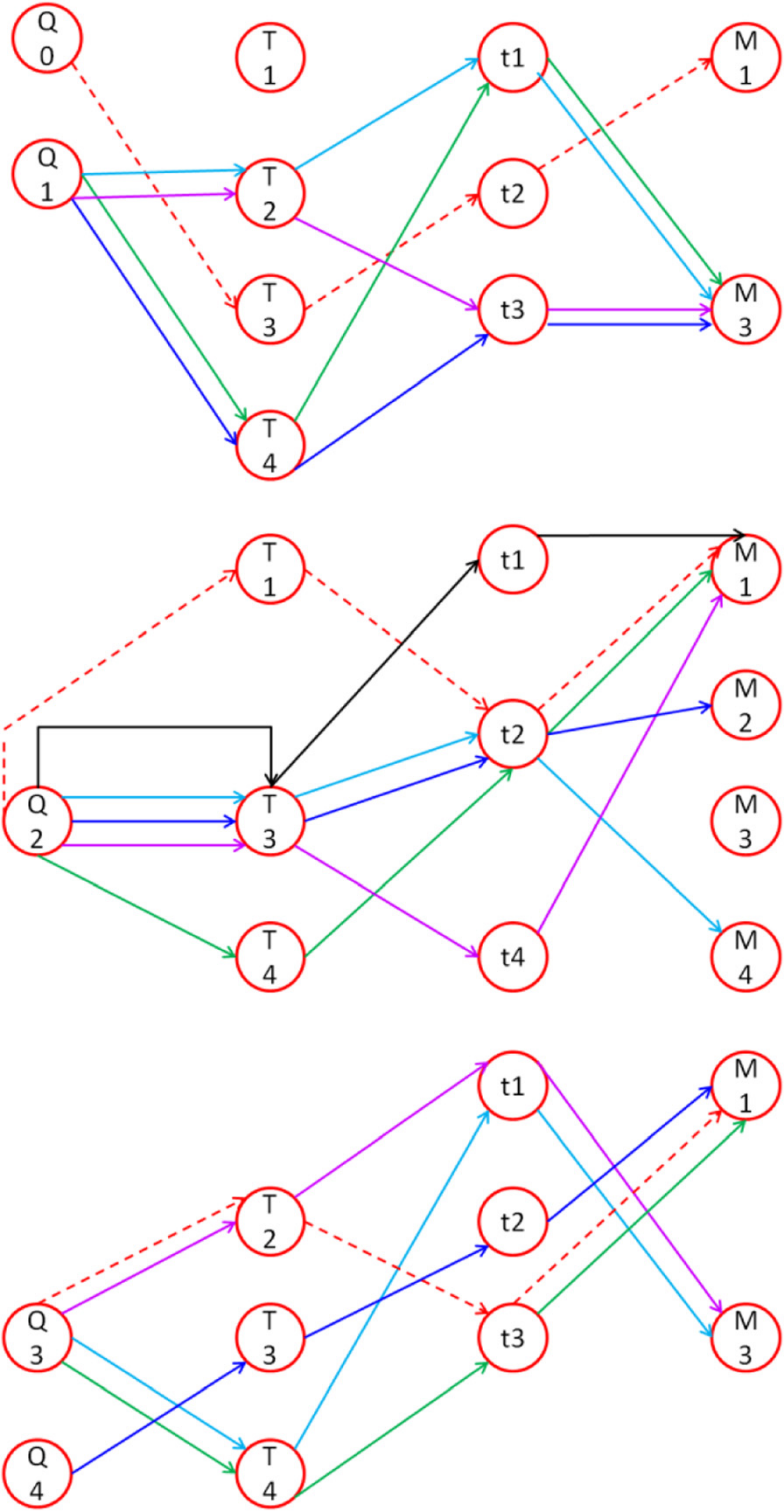
Schematic representation of parameter combination network for experimental runs

RSM was used to model the growth of ZnO nanostructures on graphene substrates using ultrasonic spray pyrolysis technique, and the optimization of the growth parameters was performed using RSM through the Box-Behnken module of response available in the Design-Expert software package (version 7.0). The Box-Behnken model was selected to correlate the four independent parameters, i.e., the precursor flow rate, molarity, substrate temperature, and injection/deposition time, which varied over four levels, with three dependent parameters or responses, i.e., the nanostructures density, shape factor, and size [22]. The formulated model was analyzed using analysis of variance (ANOVA) implemented in the Design-Expert software package. The experimental runs were determined based on the hybrid Box-Behnken model and the results of the work of Rajan and Pandit [23–25], as illustrated in Fig. 1.

Rajan and Pandit studied the effects of the ultrasonic power, frequency, and flow rate on the droplet sizes of the atomized jet. According to their work, the flow rate has a great effect on the droplet size. In our study, this conclusion was taken into consideration during the determination of the experimental runs. Thus, as observed from Fig. 1, the combinations of parameters were sorted according to the first parameter, which is a precursor flow rate, such that the investigations could focus on the effects of the flow rate of liquid precursor on the grown structure. The substrate thickness and storage conditions were kept constant and excluded from the study, respectively, to simplify the models. For film structures, the grain size was used to fit the model to determine the size response for the sake of model integrity. The diluting solvent was selected after a prior screening. The crucial ranges of the investigated parameters were obtained based on some preliminary experiments and from a literature review. These led to the values presented in Table 2, which presents the values selected for the four levels of each parameter.

**Table 2:** Selected levels for the process parameters used during experiments

Level	Flow rate (ml/min)	Temperature (°C)	Time (min)	Molarity (*M*)
Initial	0.01	135	–	–
First	0.05	210	0.5	0.05
Second	1	355	15.25	0.2
Third	8	500	30	0.4
Fourth	10	575	38	0.7

### Fitting of the Response

The averages of three runs for the independent parameters in correlation with the responses were recorded and tabulated in Table 1 following the RSM L_16_(4^4^) array for the combination of runs of the experimental parameters. The obtained results were input into the Box-Behnken model available in the Design-Expert software package (version 7.0). In the analysis procedure, the approximation of the response, *y*, was determined using a quadratic polynomial regression model as a function of the pyrolysis parameters using Eq. 1, which has linear and quadratic terms in addition to an interaction term, where *b* is the regression coefficient, *χ* is the independent parameter, and *e* is the experimental error. An automatic backward reduction of the insignificant parameters was met at a significance level of *α* ≤ 0.05. Finally, the software was used to perform ANOVA, and three-dimensional (3D) surface plots were produced.

1 $$ {y}_i={b}_0+{\displaystyle \sum {b}_i{x}_i}+{{\displaystyle \sum {b}_{jj}x}}_i^2+{\displaystyle \sum {\displaystyle \sum {b}_{ij}}}{x}_i{x}_j+e $$

## Results and Discussion

### Field-Effect Scanning Electron Microscope Results

**Fig. 2:**
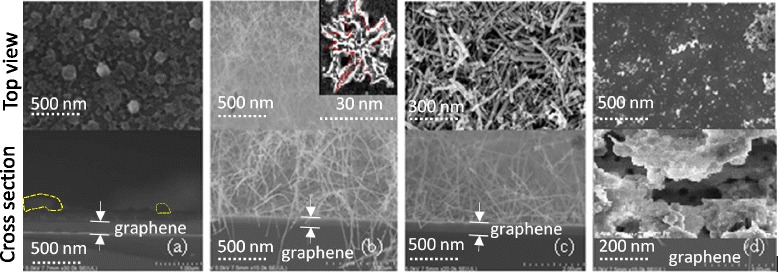
FESEM images that resulted from the **a** R4, **b** R9, **c** R7, and **d** R16 experimental runs

Figure 2 shows field-effect scanning electron microscope (FESEM) images of the grown ZnO nanostructures with different morphologies. The FESEM images were used to estimate the average diameter and size of the grown nanostructures using the AutoCad 2010 software package. The shape factor of every sample was calculated using Eq. 2, which was generalized based on the circularity factor defined by ISO 9276-6 [26].

2 $$ \varphi =\frac{24A}{{\displaystyle {p}^2}\sqrt{3}} $$

Here, φ, is the generalized shape factor of nanostructure, *A* is the area of the nanostructure, and *p* is the perimeter of the nanostructure. Figure 2a–d represents the FESEM images that resulted from the experimental runs R4, R9, R7, and R16, respectively (refer to Table 1). It can be observed from Fig. 2a that relatively large ZnO nanorods coexist in a low concentration with a ZnO film phase. Figure 2b shows a very high density of tiny ZnO nanowires with a star polygon-shaped cross-section. On the other hand, Fig. 2c shows round ZnO nanorods with a relatively large diameter and high concentration. Finally, Fig. 2d shows a porous ZnO film structure with equiaxed grains. Such results might indicate the ability of the ultrasonic-assisted spray pyrolysis to grow diverse groups of nanostructures in terms of structure shape, size, and density.

### X-Ray Diffraction (XRD) Results

**Fig. 3:**
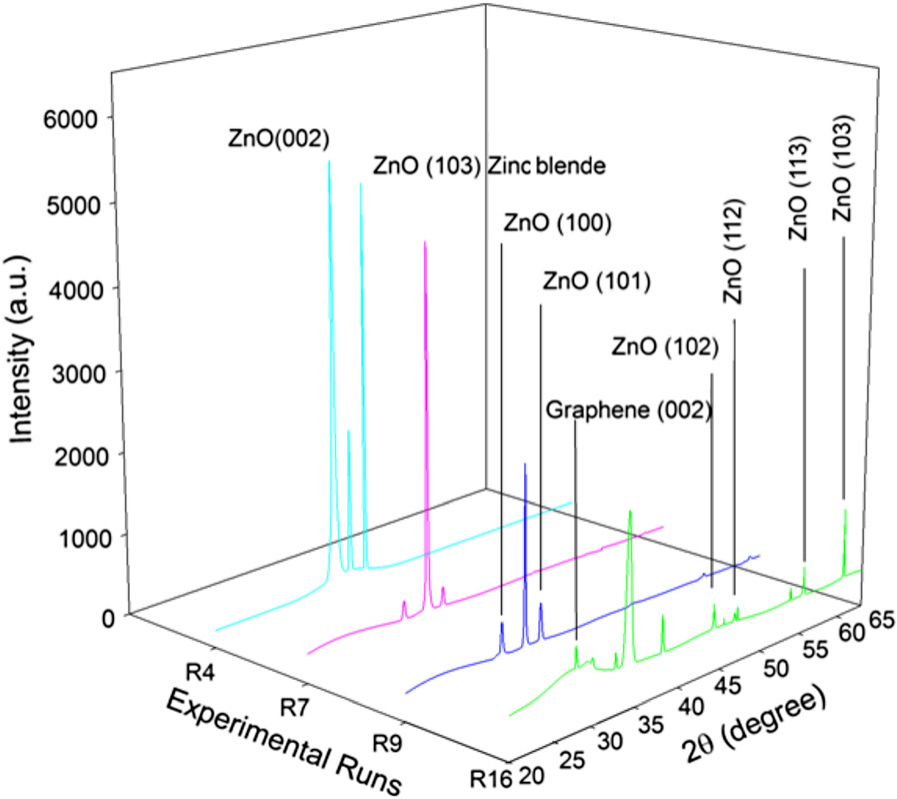
XRD patterns of the ZnO nanorods grown at experimental combination runs of R4, R7, R9, and R16

Figure 3 shows the XRD patterns of the grown ZnO nanostructures on graphene substrates. The presence of crystal planes (002), (100), and (101) indicated the growth of the hexagonal wurtzite structure of ZnO phases. The large diffraction peak detected at a 2*θ* value of 34.4^ο^ was attributed to the growth of ZnO nanorods parallel to the *c*-axis. Thus, it can be concluded for the samples from experimental runs R7 and R9 that high densities of nanorods and nanowires were achieved, respectively, because the peak corresponding to the (002) plane exhibited high intensity, whereas R4 achieved only one third of the nanorod density of the R7 sample. In contrast, the samples grown in the R16 experimental run did not exhibit any diffraction peak corresponding to the ZnO (002) plane, which indicated that the resulting structure was a ZnO thin film. The peaks corresponding to the (112) and (113) planes observed in the samples of R4 and R16 seem to suggest that the ZnO nanorods (or grains in the case of a film) might not have been perfectly hexagonal in shape. Considering the structure obtained for sample R4, the high intensity of the diffraction peak corresponding to the (103) plane zinc blende structure seemed to indicate the formation of an *a*-axis-oriented ZnO film that coexisted with the ZnO nanorod phase. The low intensities of the (100) and (110) peaks for sample R16 indicated the existence of a very low density of ZnO nanorods that coexisted with the ZnO nanofilm. For the samples of R16 and R4, the diffraction peaks corresponding to the (102) and (103) planes detected at 2θ values of 45^ο^ and 63^ο^, respectively, emphasize the existence of a polycrystalline wurtzite film structure. The obtained results clearly reveal the growth of various morphologies and phases of ZnO, which highlights the significant effects of the combination of growth parameters.

### Statistical Analysis of the Response

The averages of the three runs of the independent parameters in correlation with the responses are presented in Table 1. It is worth noting that the response data were fit by a quadratic polynomial regression equation with a significance level of *α* ≤ 0.05. The significant factors in the response equations were maintained, whereas the insignificant ones were automatically eliminated using a backward reduction method. The significances of each individual parameter and interaction parameter were estimated using a probability function analysis available in the software. Finally, the impact of the noise on the data was assessed using ANOVA, and the assessment results are tabulated and represented for every response in Table 3.

**Table 3:** Summary of ANOVA results for the fitted responses

Source	Nanorod density (*ζ*)		Shape factor (*φ*)		Nanorod size (*r*)	
	Prob > *F*	Significance	Prob > *F*	Significance	Prob > *F*	Significance
Model	<0.0001	Significance	0.019	Significance	<0.0001	Significance
*Q*	<0.0001	Significance	0.2458	Insignificance	<0.0001	Significance
*t*	<0.0001	Significance	0.1032	Insignificance	<0.0001	Significance
*M*	<0.0001	Significance	0.5334	Insignificance	<0.0001	Significance
*QM*	<0.0001	Significance	0.3644	Insignificance	<0.0001	Significance
*Qt*	NA	NA	0.0149	Significance	NA	NA
*Tt*	<0.0001	Significance	NA	NA	NA	NA
*TM*	<0.0001	Significance	0.0229	Significance	<0.0001	Significance
*tM*	<0.0001	Significance	NA	NA	<0.0001	Significance
*Q* ^2^	<0.0001	Significance	0.0559	Significance	<0.0001	Significance
*T* ^2^	<0.0001	Significance	0.0092	Significance	NA	NA
*t* ^2^	<0.0001	Significance	NA	NA	<0.0001	Significance
*M* ^2^	<0.0001	Significance	0.0252	Significance	<0.0001	Significance

*NA* not applicable

**Fig. 4:**
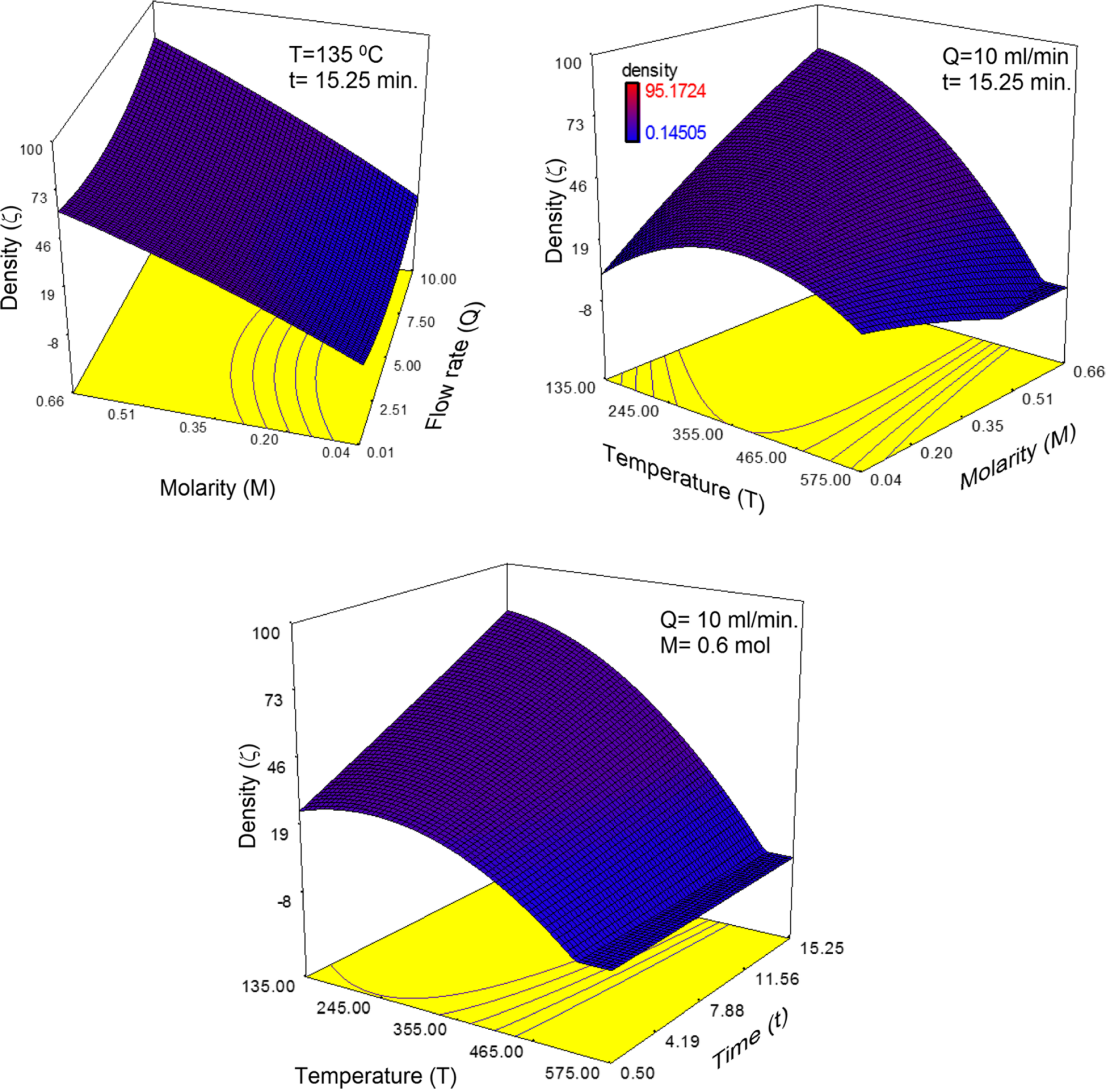
3D plots that depict the interactions between the process parameters and their impact on the nanorod density, size, and shape factor

The *F*-ratio obtained from the ANOVA is the quotient of the model mean squared divided by the error mean squared. Values of Prob > *F* of less than 0.05 suggest rejection of the null hypothesis that the coefficient of the model terms equals zero. In other words, the *F*-ratio indicates whether the terms in the model are statistically significant. It is also clear that the overall fits of the three models is significant because the Prob > *F* values of the model terms for the three responses are less than 0.05. Furthermore, by referring to the equation that relates the structure density and the process parameter, it was found that those four parameters, i.e., *Q*, *T*, *t* and *M*, had significant impacts on the response. Consequently, the response was strongly dependent on the process parameters investigated. In addition, the interactions between parameters and the quadratic forms of the parameters had significant effects on the structure density, which can be clearly observed in Fig. 4. It can be observed from Fig. 4 that a slight change in substrate temperature, injection time, or precursor molarity could result in a remarkable change in structure density. Moreover, the apparent curvatures that were captured in the 3D plots are related to the quadratic relationship of those parameters on the responses.

Regarding the equation that describes the relationship between the reaction parameters and the shape of the grown structures, it can be observed that the four process parameters did not have a direct effect on the shape of the grown structures because the Prob > F values of the linear coefficients of the model (*Q*, *T*, *t*, and *M*) are greater than 0.05. However, it is obvious that the interactions between the precursor flow rate and injection time (*Qt*) and between the precursor molarity and substrate temperature (*tM*) had significant effects on the structure shape because the value of Prob > *F* for both Qt and TM is less than 0.05.

From the equation that govern the relationship between the pyrolysis parameters and the structure size, it was found that Prob > *F* was less than 0.05 for *Q*, *T*, *t*, and *M*. Thus, the four process parameters can be said to have a direct (linear) effect on the structure size. Furthermore, it was observed that the interactions between the precursor flow rate and the precursor molarity (*QM*) had a remarkable effect on the response because the Prob > *F* value is less than 0.05. Moreover, from the Prob > *F* values for *Q*^2^, *t*^2^_,_ and *M*^2^, it was found that the precursor flow rate, injection time, and precursor molarity affected the behavior of the response in a quadratic manner.

### Nanostructure Density

The structure density, *ζ*, was determined experimentally for every combination of the parameters, as indicated in Table 1. The nanostructure density was determined as an average weight percent of ZnO nanostructures [26, 27] through five EDX spectroscopy scans performed at five different locations in the samples. The structure density was accordingly fitted to the process parameters, and the resulting response equation was formulated as indicated by Eq. 3. The regression coefficients are presented in Table 4. The statistical significance of Eq. 3 confirmed through the investigation of the impact of the noise on the response was assessed using ANOVA and is presented in Table 3.

**Table 4:** Values of the constants used in Eq. 3

Constant	Values
*c*	−114.72523
*a*_1_	−4.84837
*b*_1_	0.49626
*c*_1_	4.63634
*d*_1_	178.05071
*a*_2_ *d*_2_	2.49947
*b*_2_ *c*_2_	−8.70E−03
*b*_3_ *d*_2_	−0.41071
*c*_3_ *d*_3_	−0.060568
*a*_4_	0.47467
*b*_4_	−4.61E−04
*c*_4_	0.012818
*d*_4_	−38.4763

3 $$ \zeta =\mathrm{C}-{\mathrm{a}}_1Q+{\mathrm{b}}_1\mathrm{T}+{\mathrm{c}}_1\mathrm{t}-{d}_1M+{a}_2{d}_2QM-{b}_2{c}_2Tt-{b}_3{d}_2TM-{c}_3{d}_3tM+{a}_4{Q}^2-{b}_4{T}^2+{c}_4{t}^2-{d}_4{M}^2 $$

It can be observed from the equation that the four reaction parameters had a direct impact on the nanostructure density, especially the molarity of the liquid precursor, because the values of the linear coefficients, *a*_1_, *b*_1_, *c*_1_, and *d*_1_, are not equal to or close to zero, as indicated in Table 4. Furthermore, it is clear from the values of coefficients *a*_2_*d*_2_ and *b*_3_*d*_2_ (which are also far from zero) that the interactions between precursor flow rate and molarity, QM, and the substrate temperature and precursor molarity, TM, had a remarkable effect on the response. The values of the coefficients *a*_4_ and *d*_4_ suggest that the precursor flow rate and molarity affected the behavior of the response in a quadratic manner.

### Growth Rate and Kinetics

To validate the proposed model and the proposed Eq. 3, further comparisons of the experimental and published results were performed. A good method for such validation is to calculate the rate and activation energy using Eq. 3 and compare the calculated results with both the experimental and published results. It can be observed that Eq. 4 is a composite function in which the nanorod density is a function of the precursor flow rate and injection time, whereas the precursor flow rate itself is also a function of time. To calculate the activation rate, the rate constant should be plotted against the inverse of the substrate temperature. The temperature should also change with time; hence, the rate equation could be calculated using the time derivative of Eq. 3, as presented in Eq. 4.

4 $$ \frac{d\zeta }{dt}=\frac{\partial \zeta }{\partial Q}\cdot \frac{dQ}{dt}+\frac{\partial \zeta }{\partial T}\cdot \frac{dT}{dt} $$

Here, *dT*/*dt* is taken to be 10 °C/min, which is equal to the heating rate used in the experiments. *∂ζ/∂Q* and *∂ζ/∂T* can be calculated by taking the partial derivatives of Eq. 3 with respect to the flow rate and temperature, respectively, as shown in Eqs. 5 and 6.

5 $$ \frac{\partial \zeta }{\partial Q}=-4.84837 + 2.49947M + 0.94934Q $$

6 $$ \frac{\partial \zeta }{\partial T}=0.49626-0.0087t-0.41071M-0.000922T $$

7 $$ Q={\left(\frac{\sigma t}{\rho}\right)}^{-0.151}\cdot {\left[17.24138.{d}_p.{\left(\frac{\pi \sigma {t}^2}{\rho}\right)}^{-0.33}.{\displaystyle {I_N}^{0.0277}}.{\displaystyle {(Oh)}^{-0.192}}\right]}^{5.845} $$

**Fig. 5:**
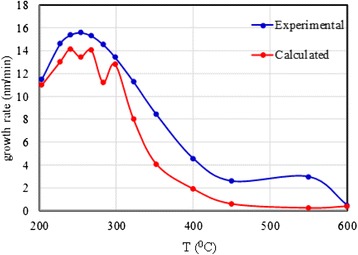
The relation between the growth rate and substrate temperature

For the sake of the accuracy of the calculation of *dQ/dt*, the flow rate of the atomized precursor was calculated using the empirical formula presented in the work by Rajan and Pandit [25], which relates the jet droplet size to the physical properties of the liquid precursors and the ultrasonic power. Their equation is rewritten and presented in Eq. 7. The derivative of Eq. 7 with respect to time was determined and substituted in Eq. 4, and the growth rate was then plotted against the substrate temperature, as shown in Fig. 5.

It can be observed from Fig. 5 that the calculated results exhibit the same trend as the experimental results. The fluctuations observed in the calculated results curve can be attributed to the nature of the Taylor series polynomial equation given by Eq. 1 used for response fitting. According to the reaction specified by Eq. 8, the reaction seemed to exhibit a first-order behavior. Thus, Eq. 4 can be substituted in the rate law, as indicated by Eq. 9.

8 $$ Zn{\left({C}_5{H}_7{O}_2\right)}_2.{H}_2O+2{C}_2{H}_6O\to ZnO+2{C}_7{H}_{14}{O}_3 $$

9 $$ \frac{d\zeta }{dt}=K\left[A\right]\left[B\right] $$

**Fig. 6:**
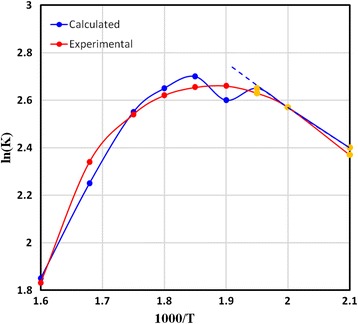
Arrhenius plot

Here, [*A*] and [*B*] are the concentrations of Zn(*acac*_2_)_2_ and ethanol, respectively, which are equal to the negative value of the concentration of ZnO and C_7_H_14_O_3_, respectively. *K* is the reaction constant, which can be directly calculated from Eq. 9 at any step. Consequently, an Arrhenius plot can be plotted for ln(*K*) versus the inverse of substrate temperature, as shown in Fig. 6. The slope of the resulted curve was used to determine the activation energy (*E*_a_) following the Arrhenius equation. The activation energy calculated from the simulation data was found to be approximately 15.13 kJ/mol, which is in good agreement with our experimental results (14.53 kJ/mol) and previously published results (14.47 kJ/mol) [1]. These results seem to validate the model and emphasize the reliability of Eqs. 3 and 4 to describe the phase transformations and growth rates in term of process parameters.

### Nanorod Shape Factor

The shape factor, *φ*, of the grown nanorods was determined experimentally for every combination of parameters indicated in Table 1. The nanostructures shape factor was determined by averaging the calculated value of *φ* using Eq. 2 for five FESEM scans performed at five different locations in the sample. The nanostructures shape factor could be accordingly fit to the process parameters, and the resulting response equation was formulated, as given by Eq. 10. The regression coefficients are summarized in Table 5.

**Table 5:** Values of the constants used in Eq. 10

Constant	Values
*c*	0.69633
*a*_1_	−0.017417
*b*_1_	−0.00155153
*c*_1_	0.12826
*d*_1_	0.40719
*a*_2_ *c*_2_	−0.00658285
*a*_3_ *d*_2_	0.039737
*b*_2_ *d*_3_	0.00293418
*a*_4_	0.00686594
*d*_3_	−0.00523141
*d*_4_	−2.11193

10 $$ \varphi =\mathrm{c}+{\mathrm{a}}_1Q+{\mathrm{b}}_1\mathrm{T}+{\mathrm{c}}_1\mathrm{t}+{d}_1M+{a}_2{c}_2Qt+{a}_3{d}_2QM+{b}_2{d}_3TM+{a}_3{Q}^2+{b}_3{T}^2+{d}_4{M}^2 $$

It can be understood from Eq. 10 that the four process parameters had a direct effect on the shape of the grown nanorods, especially the injection time and molarity of the liquid precursor (the latter had the highest linear coefficients, *a*_1_ = 0.12826 and *d*_1_ = 0.40719, respectively). However, by comparing such observations to the results of the ANOVA presented in Table 3, the results show that all of the four parameters (*Q*, *T*, *t*, and *M*) did not have a direct statistically significant effect on the response. However, it is obvious that the interactions between molarity and the precursor flow rate produced a significant effect on the response because the value of coefficient *a*_3_*d*_2_ was significant, as indicated in Table 5.

**Fig. 7:**
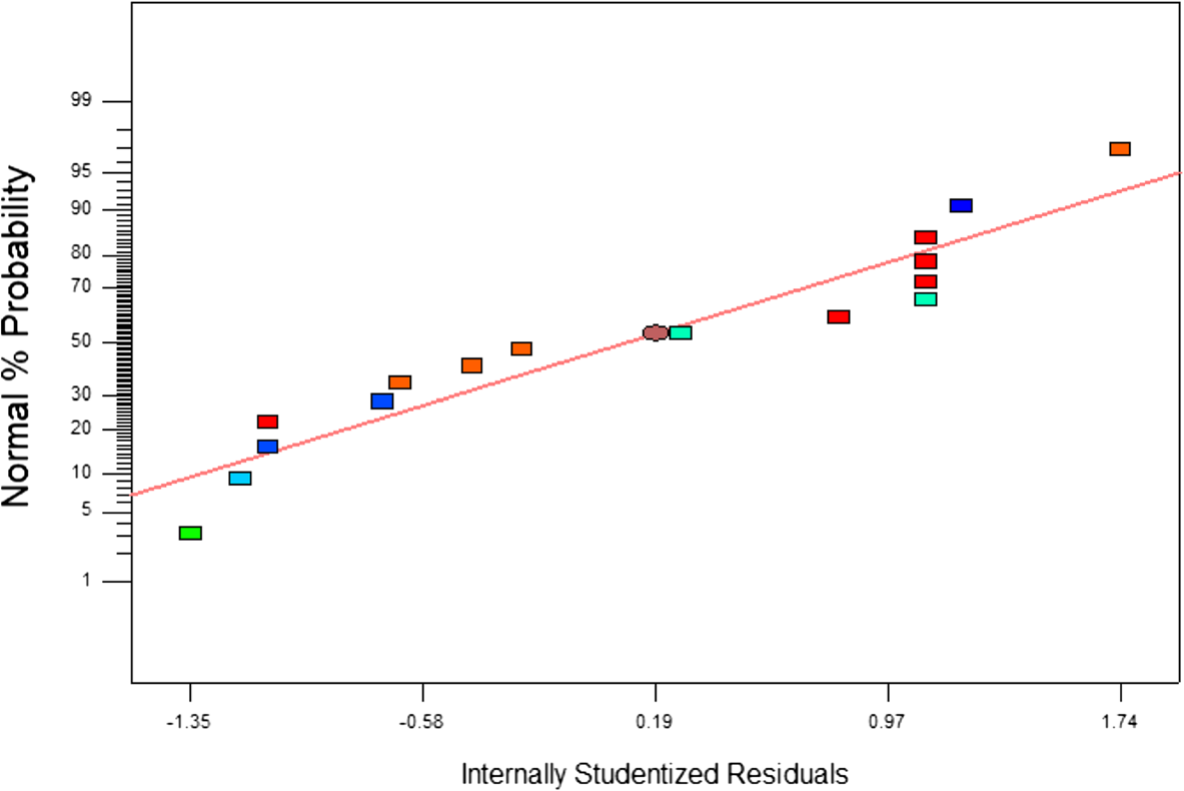
Normal probability plot for nanorod shape factor fitting

Statistical tools such as ANOVA and normal plots of residuals were used to investigate the significance of Eq. 10 and robustness of the model further. To our knowledge, there is no published report regarding the evaluation of the structure shape factor. The impact of the noise on the response was gauged using ANOVA and is presented in Table 3. The normal probability plot shown in Fig. 7 is a graphical tool that quantifies the functional departure of the results from normality. The normal probability plot is based on the experimentally determined shape factor, the residuals from model fitting, and the estimated parameters. It can be observed that the normal percentage (%) probability is plotted versus the internally studentized residuals, where the trend of the results is be represented by a straight line. Thus, it is clear that the data were normally distributed, i.e., there was no departure from normality, and no obvious irregularity occurred during fitting. This result indicates a high robustness of the model in general and of Eq. 10 in particular.

**Fig. 8:**
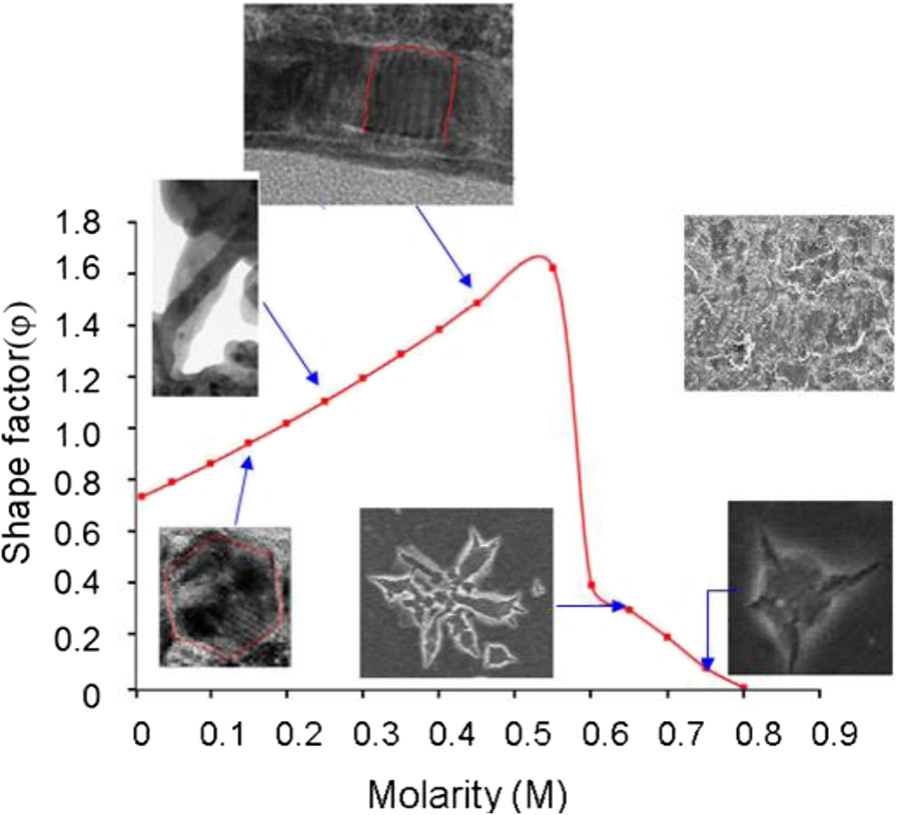
The relationship between the shape factor of the nanorods and the molarity of precursor at constant injection time (*t* = 15.25 min), constant temperature (*T* = 134 °C), and constant precursor flow rate (*Q* = 8 ml/min)

According to the previously discussed ANOVA results for Eq. 10, the equation was solved for the full range of precursor molarity (0.05–0.8 M) at constant injection time (*t* = 15.25 min), constant substrate temperature (*T* = 134 °C), and constant precursor flow rate (*Q* = 8 ml/min); the results are summarized in Fig. 8. The quadratic effect of the molarity can be clearly observed from the trend in the results. Moreover, the iterations proved to be helpful for the detection of the nanorod shapes, as observed in Fig. 8. For instance, the phase transition point from a nanorod to granular thin film was detected at a precursor molarity of approximately 0.55 M. In fact, at a precursor molarity equal to 0.50 M, the grown structure was found to be a mixture of thin film and nanorods in a composite form, whereas at a precursor molarity of 0.6 M, the grown structure was found to be solely thin film. Such findings seem to be helpful for the sake of material design.

**Fig. 9:**
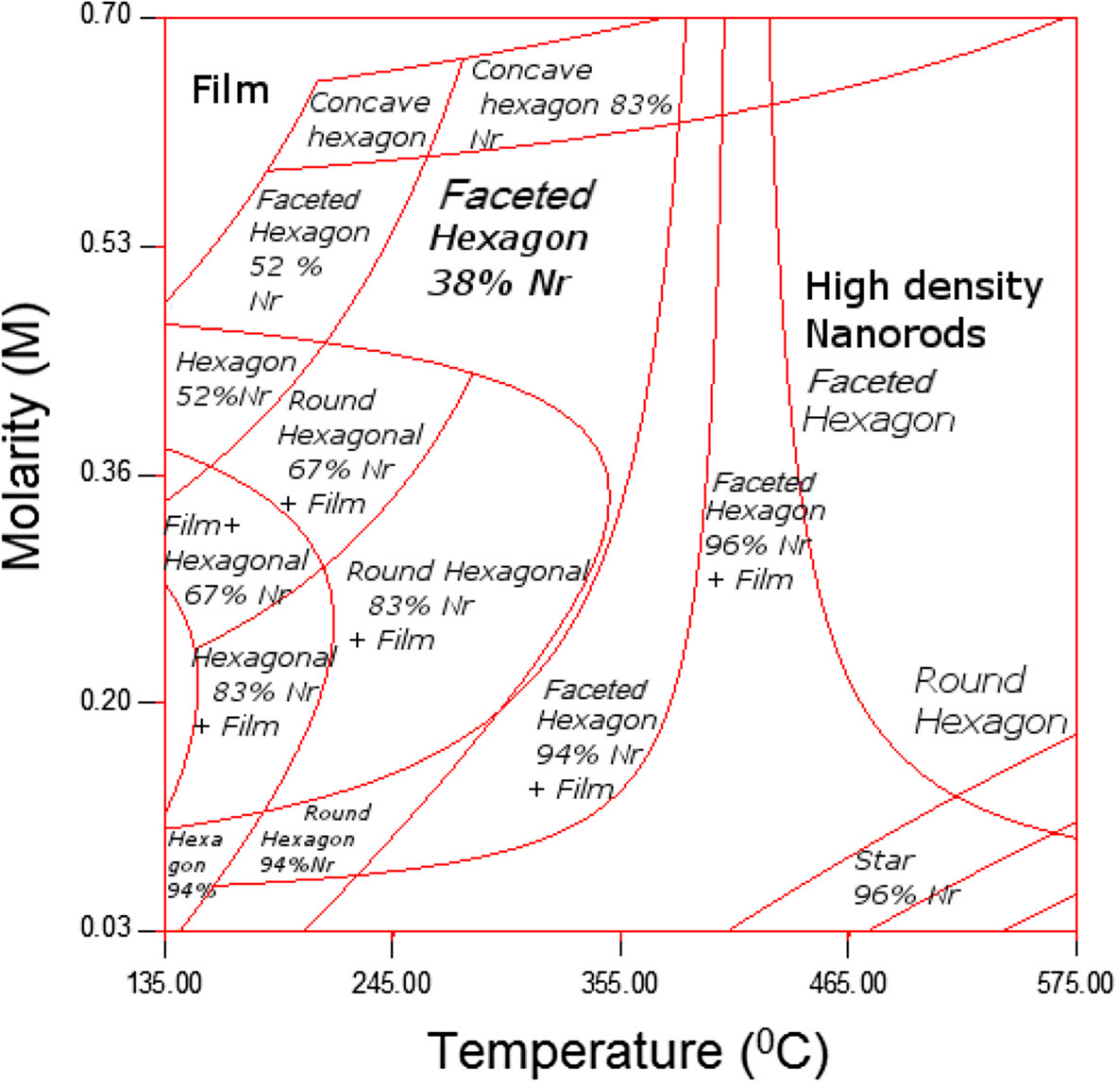
Phase transformation diagrams depicting the relationship between the precursor molarity and the substrate temperature, structure density, and structure shape

As a result, it was found that by solving Eqs. 3 and 10 together for a full range of process parameters, it was possible to plot the phase transformation diagram, as shown in Fig. 9. It can be observed that the precursor molarity is plotted versus the substrate temperature, structure density, and structure shape. The red curves determine the phase change borders and the phase transformation affinity. For instance, for substrate temperature ranging from 134 to 200 °C and precursor molarity ranging from 0.42 to 0.7 M, the grown structure slowly transformed from a nanorod with a faceted hexagonal profile with 52 % nanorod density to a film with hexagonal grains. For the same molarity range but a temperature range of 201 to 355 °C, the phase transformation occurred rapidly, as is indicated by the rate of change in the slope of the transformation curve. In contrast, for a substrate temperature ranging from 455 to 575 °C and precursor molarity ranging from 0.1 to 0.7 M, the grown structure rapidly transformed from star-shaped nanorods with 96 % phase density to a high density of nanorods with faceted hexagonal profiles. Such an empirically driven phase transformation diagram can enable tailoring various families of ZnO/graphene structures with various morphologies without the need for further experimental work except for validation.

**Fig. 10:**
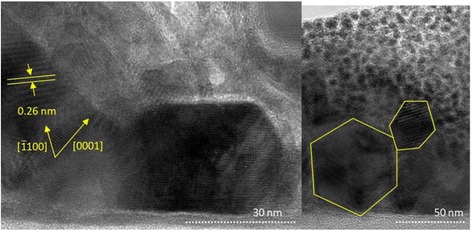
TEM images of nanorods grown at a substrate temperature of 240 °C, precursor molarity of 0.2 M, flow rate of 0.05 ml/min, and injection time of 10 min

For the sake of precise investigation of the effect of the pyrolysis parameter on the shape of nanorods, transmission electron microscopy (TEM) scanning was performed to validate the results of the optimization. The obtained TEM images are shown in Fig. 10. The nanorods shown in the images were grown at a substrate temperature of 240 °C, precursor molarity of 0.2 M, flow rate of 0.05 ml/min, and injection time of 10 min. It can be observed that the spacing between lattice planes was approximately equal to 0.26 nm along the $\left[\overline{1}\;100\right]$ direction, which indicates slightly inclined nanorods. The crystalline single layer of graphene appears to be non-deformed despite being damaged during dual-beam preparation of the sample. Furthermore, the TEM images show the different true-hexagon and faceted-hexagon shapes of the obtained nanorods. This observation strongly validates the optimization results.

The nanorods and nanowire size, *r*, was determined experimentally for every combination of parameters, as indicated in Table 1. The size of the nanostructure (nanorods and nanowire) was determined by calculating the average diameter of a circle that was a tangent to the outer profile of the nanorods using three FESEM images taken at five different locations in the sample. The diameter of the nanostructure was accordingly fitted to the process parameters, and the resulting response equation was formulated as indicated by Eq. 11. The regression coefficients are presented in Table 6.

**Table 6:** Values of the constants used in Eq. 11

Constant	Values
*c*	4.132878115
*a*_1_	1.340365861
*b*_1_	0.076423887
*c*_1_	0.228005904
*d*_1_	20.37838155
*a*_2_ *d*_2_	−0.653868789
*c*_2_ *d*_3_	−0.08490782
*b*_2_ *d*_4_	0.00121007
*a*_3_	−0.089368492
*c*_3_	0.003004526
*d*_5_	−7.303685998

11 $$ r=\mathrm{C}+{\mathrm{a}}_1Q+{\mathrm{b}}_1\mathrm{T}+{\mathrm{c}}_1\mathrm{t}+{d}_1M+{a}_2{d}_2QM+{c}_2{d}_3Mt+{b}_2{d}_4TM+{a}_3{Q}^2+{c}_3{t}^2+{d}_5{M}^2 $$

It can be understood that the four process parameters (*Q*, *T*, *t*, and *M*) had a direct (linear) effect on the size of the grown nanorods, especially the molarity of the liquid precursor because it had the highest linear coefficient, *d*_1_ = 20.37. This observation is valid according to the results of the ANOVA, which are presented in Table 3. Furthermore, it can be observed that the interactions between the precursor flow rate and precursor molarity had a remarkable effect on the response because the value of coefficient *a*_2_*d*_2_ listed in Table 6 is 0.65. Moreover, the value of the coefficient *d*_5_ indicates that precursor molarity affected the behavior of the response in a quadratic manner. The growth rate of a single nanorod could be directly calculated by taking the partial derivative of *r* with respect to time, as indicated by Eq. 12.

12 $$ \frac{\partial r}{\partial t}=0.228+0.003t-0.0849M $$

**Fig. 11:**
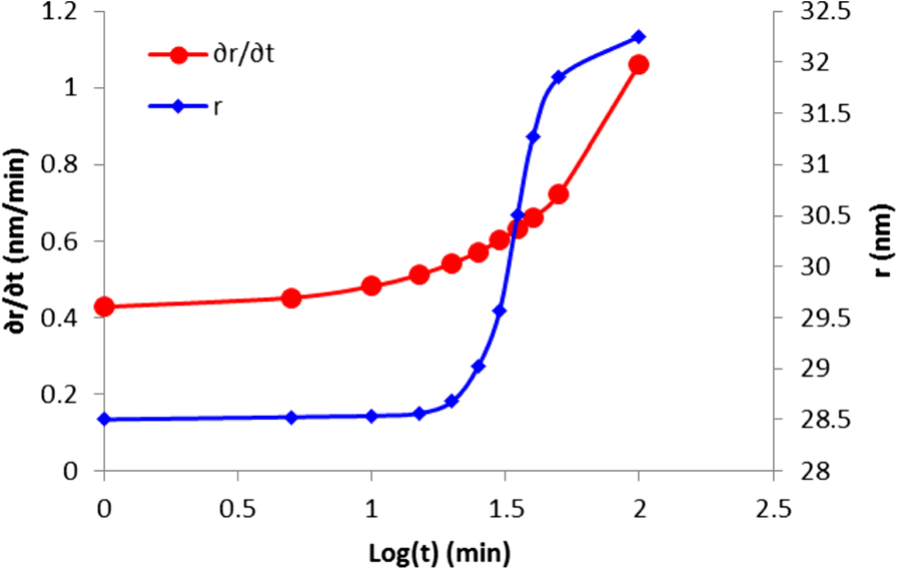
Relationships between the calculated growth rate and the nanorod size and injection time

The calculated rate was plotted against time and nanorod size, as shown in Fig. 11. It was found that the size and growth rates are directly proportional; this trend is similar to that of the Johnson-Avrime-Mehl growth model [28–31]. It is clear that after 15 min (log(*t*) = 1.17), the changing rate of nanorod size with respect to time increased dramatically, thus leaving a narrow window (25 min) for controlling the nanorod size by controlling the injection time. Thus, the first 15 min of deposition were insufficient for controlling the sizes of the grown nanorods via controlling the injection time. However, the next 25 min could enable adequate control of the nanorod growth. Furthermore, the growth rates presented in Fig. 11 are consistent with the published results [32].

Finding an equation that can describe the relationship between the size of the grown nanorods and the size of the droplets of liquid precursor spray could be helpful. To investigate this relation, the injection flow rate should be considered to be approximately equal to the droplet flow rate. Thus, for the sake of accuracy, Eq. 7 was used to calculate the droplet size, *d*_p_, at different values for the flow rate according to Eq. 11. Moreover, the rate of change in the nanorod size with respect to the flow rate (*∂r/∂Q*) could be formulated as indicated by Eq. 13.

13 $$ \frac{\partial r}{\partial Q}=1.34-0.653M-0.1787Q $$

Equation 13 could be rewritten as indicated by Eq. 14 by substituting Eq. 7 into Eq. 13.

14 $$ \frac{\partial r}{\partial Q}=1.34-0.653M-0.1787\left({\left(\frac{\sigma }{\rho f}\right)}^{-0.151}\cdot {\left[17.24138.{d}_p.{\left(\frac{\pi \sigma }{\rho {f}^2}\right)}^{-0.33}.{\left({I}_N\right)}^{-0.0277}.{\displaystyle {(Oh)}^{-0.192}}\right]}^{5.845}\right) $$

**Fig. 12:**
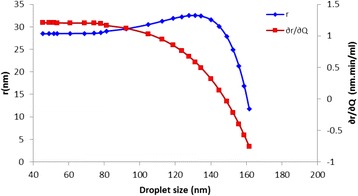
The relationship between the droplet size of the atomized precursor and the size of the resulting nanorods

Rearranging Eq. 14 in such a manner enables the construction of the plot shown in Fig. 12. The relation between the droplet size and the size of the resulting nanorods exhibited a second-order polynomial trend. It can be observed that as the droplet size increased, the nanorod size increased. This result can be attributed to the increase in the number of reacting species and the reaction surface on the substrate surface. This behavior continued until the yield point was reached at a droplet size of 135 nm, after which the nanorod diameter decreased rapidly. This result can be explained by considering Eqs. 3, 7, and 11. In these equations, the droplet size increases proportionally with the flow rate. Higher flow rates resulted in lower density of nanorods but higher coverage of the film phase.

## Conclusions

Various types of nanostructures thin film were grown on graphene substrates using ultrasonic assisted spray pyrolysis in the presence of alcohol. The process parameters were determined according to the L_16_(4^4^) array generated via RSM. The impacts of the process parameters on the nanostructure density, size, and shape were investigated. The relationships between the process parameters and the responses were modeled and optimized using a statistical approach. A set of 3D plots and phase transformation diagrams were generated. These results enable the selectivity of parameters corresponding to a certain nanostructure density, size, and shape of interest. Furthermore, the growth rate and kinetics were studied, and it was found that nanostructure density and size were influenced by the precursor molarity and flow rate in a quadratic manner. The nanostructure shape was found to be not only molarity- and flow rate-dependent but also temperature-dependent. This work has successfully formulated several important equations and graphs that are able to describe the relationship between the droplet sizes of the atomized precursors and the sizes of the grown nanostructures.
